# Closure in the Time of COVID-19

**DOI:** 10.5811/westjem.2020.5.48050

**Published:** 2020-05-22

**Authors:** Mark A. Brubaker

**Affiliations:** Hackensack University Medical Center, Department of Emergency Medicine, Hackensack, New Jersey

“I was able to tell him that I am naming my son after him,” she confessed. I found myself more emotional than anticipated by this stranger’s news. I met Sarah (name has been changed) a week prior to this phone call, because, as a doctor in the emergency department, I had treated her in our “COVID tent.” Like the majority of patients battling COVID-19, she was medically stable and safely discharged home to endure the path toward physical recovery.

However, before her discharge, Sarah pulled two Ziploc bags out of her purse. Each was neatly labeled with a name, holding a cell phone and charger inside. One bag had the name of her father; the other bag had the name of her grandfather. They were not as lucky in their battles against the COVID-19 virus, as they were currently fighting for their lives in our hospital. Sarah’s only request was to find a way to get these phones to them, so that she and the rest of her family could hear their voices again.

Our hospital, like most facing this pandemic, has a very restricted visitor policy in order to “flatten the curve.” Families are unable to sit at the bedside of their sick loved ones. Time that is often spent comforting one another, sharing information, or even “getting affairs in order” is now stolen away by the intensely isolating conditions required for managing this contagion. I was at the end of my shift, and therefore a bit more free to deliver these phones to her family’s nurses. This was a small and simple act, compared to other acts of heroism performed at the hospital each day, yet I could not predict how important this technological connection would be to the family at large.

I called Sarah a week after our ED visit to check on her, since she was six months pregnant and fighting her own COVID infection. She relayed that she was recovering well. She then expressed her gratitude for the delivery of the cell phones. With them, she was able to have many deep conversations with her father over the subsequent three days. “I got to tell him that I love him. I got to tell him that I and my [unborn] baby are okay. And I got to tell him that I will be naming my son after him.”

Sarah went on to tell me that her father died that morning. She lost her father, but her last memories of him are filled with meaningful conversations. Sarah expressed how much that meant for her to have closure.

Even after her father died, her grandfather remained in the hospital, slowly losing his battle against COVID. As his body continued to falter, they were able to bring him home on hospice, to be with the family before his death. He was able to come home because a phone was plugged in and voices connected.

This simple act of delivering these cell phones allowed Sarah to bring some sort of closure during these tragic and sudden losses. As healthcare workers, we are on the front lines fighting with our patients against COVID-19. Most of our patients do well. Unfortunately, many do not survive. However, we can help every patient and family to retain their humanity in this overwhelming time. Every little thing we do makes a difference in the lives of our patients and their families.

The COVID-19 pandemic has forced clinical providers to adapt nearly every part of our practice to provide medical care for patients, and we also need to evolve how we support their loved ones coping with the multiple levels of separation. As frontline providers, when we treat family members in the ED then we have the chance to help them make connections with their family upstairs. While we do not always see the downstream effects, even the small acts can make a major positive impact for our patients. We need to focus on helping our patients and families to connect in these dire times. That connection can allow the patients, families, and even providers, to find closure and meaning, even if cure and simple physical proximity are unattainable.

